# Associations of TSH, free T3, free T4, and conversion ratio with incident hypertension: results from the prospective Brazilian Longitudinal Study of Adult Health (ELSA-Brasil)

**DOI:** 10.20945/2359-4292-2023-0301

**Published:** 2024-05-10

**Authors:** Marina Gabriela Birck, Carolina C. P. S. Janovsky, Alessandra Carvalho Goulart, Vandrize Meneghini, Bianca de Almeida Pititto, José Augusto Sgarbi, Patrícia de Fátima dos Santos Teixeira, Isabela M. Bensenor

**Affiliations:** 1 Universidade de São Paulo Hospital Universitário Centro de Pesquisas Clínicas e Epidemiológicas São Paulo SP Brasil Centro de Pesquisas Clínicas e Epidemiológicas, Hospital Universitário, Universidade de São Paulo, São Paulo, SP, Brasil; 2 Universidade Federal de São Paulo Escola Paulista de Medicina Departamento de Medicina São Paulo SP Brasil Serviço de Endocrinologia, Departamento de Medicina, Escola Paulista de Medicina, Universidade Federal de São Paulo, São Paulo, SP, Brasil; 3 Universidade de São Paulo Faculdade de Saúde Pública Departamento de Epidemiologia São Paulo SP Brasil Departamento de Epidemiologia, Faculdade de Saúde Pública, Universidade de São Paulo, São Paulo, SP, Brasil; 4 Universidade Federal de São Paulo Escola Paulista de Medicina Departamento de Medicina Preventiva São Paulo SP Brasil Departamento de Medicina Preventiva, Escola Paulista de Medicina, Universidade Federal de São Paulo, São Paulo, SP, Brasil; 5 Unidade de Endocrinologia Faculdade de Medicina de Marília Marília SP Brasil Unidade de Endocrinologia, Faculdade de Medicina de Marília, Marília, SP, Brasil; 6 Universidade Federal do Rio de Janeiro Faculdade de Medicina Rio de Janeiro RJ Brasil Faculdade de Medicina, Universidade Federal do Rio de Janeiro, Rio de Janeiro, RJ, Brasil

**Keywords:** Thyroid function tests, thyroid hormones, hypertension, incidence, cohort studies

## Abstract

**Objective:**

To evaluate the association of TSH, free T3 (FT3), free T4 (FT4), and conversion (FT3:FT4) ratio values with incident hypertension.

**Materials and methods:**

The study included data from participants of the ELSA-Brasil study without baseline hypertension. Serum TSH, FT4 and FT3 levels, and FT3:FT4 ratio values were assessed at baseline, and incident hypertension (defined by blood pressure levels ≥ 140/90 mmHg) was estimated over a median of 8.2 years of follow-up. The risk of incident hypertension was evaluated considering a 1-unit increase in TSH, FT4, FT3, and conversion ratio values and after dividing these variables into quintiles for further analysis using Poisson regression with robust variance. The results are presented as relative risks (RR) and 95% confidence intervals (CIs) before and after adjustment for multiple variables.

**Results:**

The primary analysis incorporated data from 5,915 euthyroid individuals, and the secondary analysis combined data from all euthyroid individuals, 587 individuals with subclinical hypothyroidism, and 31 individuals with subclinical hyperthyroidism. The rate of incident hypertension was 28% (95% CI: 27%-29.3%). The FT4 levels in the first quintile (0.18-1.06 ng/dL) were significantly associated with incident hypertension (RR: 1.03, 95% CI: 1.01-1.06) at follow-up. The association between FT4 levels in the first quintile and incident hypertension was also observed in the analysis of combined data from euthyroid individuals and participants with subclinical thyroid dysfunction (RR: 1.04, 95% CI: 1.01-1.07). The associations were predominantly observed with systolic blood pressure levels in euthyroid individuals. However, in the combined analysis incorporating euthyroid participants and individuals with subclinical thyroid dysfunction, the associations were more pronounced with diastolic blood pressure levels.

**Conclusion:**

Low FT4 levels may be a mild risk factor for incident hypertension in euthyroid individuals and persons with subclinical thyroid dysfunction.

## INTRODUCTION

Thyroid hormones affect cardiac output, systemic vascular resistance, blood volume, heart rate, and cardiac contractility (1,2). Levels of TSH, free T4 (FT4), and free T3 (FT3) have been associated, albeit inconsistently, with hypertension and changes in systolic (SBP) and diastolic blood pressure (DBP) levels, indicating a possible common mechanism between these hormones and hypertension (3,4). Indeed, a recent meta-analysis reported a dose-response relationship between TSH levels and hypertension risk (4).

Cross-sectional studies have reported positive associations between FT4 levels and DBP (5-7) and, less frequently, SBP (7,8) in euthyroid individuals. Other studies have reported associations between serum TSH levels and high blood pressure values (5,9,10), although this finding has not been confirmed in all studies (11,12). Additionally, prospective studies have reported a positive association between FT4 and DBP levels (13,14). While TSH levels have been associated with changes in DBP levels in women (15), some studies have reported no association between TSH levels and incident hypertension (16,17). Conflicting data have also been observed regarding serum FT3 levels, in which positive (6,8) and negative (18) associations with hypertension have been verified. The relationship between thyroid function and hypertension in individuals with subclinical thyroid dysfunction has been studied less, but results from cross-sectional (19-22) and prospective studies (17) have been inconsistent.

The conversion (FT3:FT4) ratio, which represents the conversion of T4 to T3, is emerging as a more precise and feasible indicator of thyroid hormone metabolic variability and is a stronger prognostic marker than FT3 or FT4 alone (23). Indeed, recent studies have found the FT3:FT4 ratio to be an independent predictor of cardiovascular disease risk and mortality (23).

Thus, the aim of the present study was to evaluate the association of TSH, FT4, FT3, and FT3:FT4 ratio values with incident hypertension in data collected prospectively as part of the Brazilian Longitudinal Study of Adult Health (ELSA-Brasil), which includes a multiethnic highly admixed cohort different from the populations analyzed in previous studies. Notably, this is the first study evaluating the association between the FT3:FT4 ratio and the incidence of hypertension.

## MATERIALS AND METHODS

ELSA-Brasil is a prospective multicenter cohort study including 15,105 civil servants aged 35-74 years from six cities in Brazil. Detailed information about the study has been published previously (24-27). Baseline (2008-2010) and follow-up (2017-2019) assessments in the study included a broad set of questionnaires, clinical variables, and laboratory tests obtained by trained staff according to standardized protocols and under rigorous supervision (24,26-28). The protocol of the study was approved by each institutional ethics committee (approval numbers 0017.1.069.000-06BA, 08109612.7.20035060ES, 0186.1.203.000-06MG, 0017.1.069.000-06RS, 0058.0.011.000-07RJ, and 0016.1.198.000-06SP for the baseline assessments and 30614714.8.1001.5030BA, 08109612.7.20035060 ES, 0186.1.203.000-06MG, 48608515.5.1001.5327 RS, 56021516.0.1001.5240RJ, and 08109612.7.1001. 0076SP for the follow-up assessments). The study was conducted in accordance with the Declaration of Helsinki, and all participants signed an informed consent.

### Thyroid function and hypertension

Venous blood samples were drawn in the morning (between 6:30 and 9:00 am) after overnight fast. Serum levels of TSH (normal range [NR] 0.40-4.00 mIU/L), FT4 (NR 0.93-1.70 ng/dL), and FT3 (NR 0.20-0.44 ng/dL) were determined using third-generation immunoenzymatic assay (Roche Diagnostics, Mannheim, Germany). The primary analysis incorporated participants who were euthyroid, as determined by a serum TSH level of 0.40-4.00 mIU/L and no history of use of levothyroxine or antithyroid medications. A secondary analysis was performed including participants with subclinical hyperthyroidism (TSH level below 0.40 mIU/L and FT4 level 0.93-1.70 ng/dL) and subclinical hypothyroidism (TSH level above 4.00 mIU/L and FT4 level 0.93-1.70 ng/dL) (26). Participants with overt hyperthyroidism or hypothyroidism were excluded from the analysis, along with those using medications with effects on thyroid function (*e.g.*, amiodarone, biotin, carbamazepine, carbidopa, furosemide, haloperidol, heparin, levodopa, lithium, metoclopramide, phenytoin, propranolol, primidone, rifampicin, systemic corticosteroids, and valproic acid).

Hypertension was defined by SBP ≥ 140 mmHg, DBP ≥ 90 mmHg, or use of medication to treat high blood pressure (29). Three blood pressure measurements were performed at the research center, and the average of the second and third measures was documented as a "casual blood pressure" level. Individuals with hypertension at baseline were excluded.

**Figure 1:**
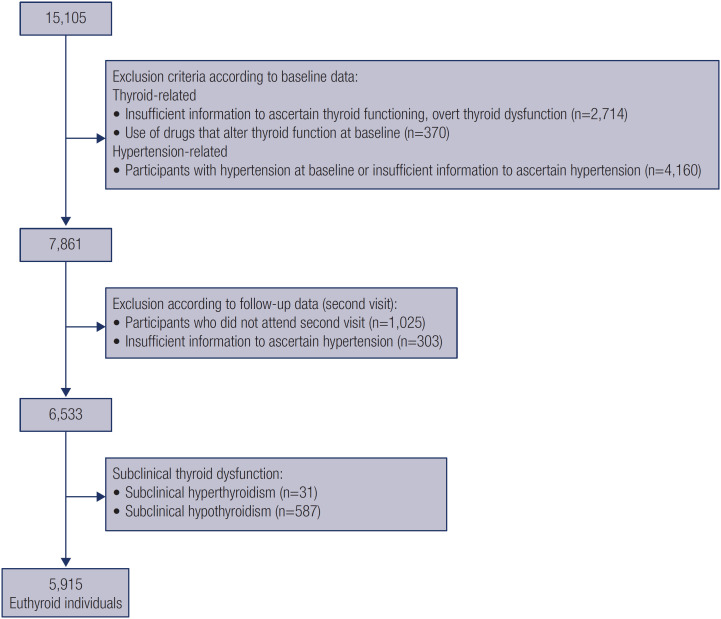
Flowchart of cohort selection.

After applying the selection criteria, a total of 5,915 participants with euthyroidism and 618 with subclinical thyroid dysfunction from the original cohort were included in the analysis (Figure 1).

### Other baseline variables

Questionnaires collected information on age, education level (less than high school, high school and some college, complete college or more), average monthly net family income (<$1,245, $1,245-3,319, and ≥ $3,320, represented in US dollars, considering a conversion rate of 2 Brazilian reais to 1 US dollar defined at study baseline), self-reported race (white, mixed, black, Asian, or indigenous), coverage by private health insurance plan (yes or no), and smoking and alcohol consumption (never, past, or current for each). Physical activity during leisure time was assessed using the International Physical Activity Questionnaire, and the participants were categorized according to their responses as inactive, insufficiently active, or active (30,31). Medication use during the last 2 weeks before the interview was recorded. Standard techniques were applied for the calculation of body mass index (BMI) and measurement of waist circumference (32). Dyslipidemia was defined as a low-density lipoprotein (LDL) cholesterol level ≥ 130 mg/dL or the use of lipid-lowering medication (33). Total cholesterol, high-density lipoprotein (HDL) cholesterol, and triglyceride (glycerol phosphate peroxidase method) levels were measured using an enzymatic colorimetric assay (**ADVIA 1200,** Siemens, Deerfield, USA). Levels of LDL cholesterol were calculated using the Friedewald equation, except for cases in which triglyceride levels were > 400 mg/dL, when an enzymatic colorimetric assay (ADVIA 1200) was used.

### Statistical analysis

Categorical variables are described as absolute numbers and proportions, and continuous variables as mean (standard deviations). Normality was tested using the Kolmogorov-Smirnov goodness-of-fit test.

The analyses were performed considering data collected at baseline and a mean 8.2 years follow-up. The primary analysis was conducted in euthyroid participants considering a 1-unit increase in TSH, FT4, FT3, and conversion ratio values, and with these variables divided into quintiles. A secondary analysis was performed considering euthyroid individuals and participants with subclinical thyroid dysfunction together.

The risk of incident hypertension was assessed using Poisson regression models with robust variance using TSH, FT4, FT3, and FT4:FT3 ratio values divided into quintiles and shown as relative risks (RRs) with 95% confidence intervals (95% CIs). The third quintiles were used as references to verify the occurrence of a U-shaped curve, in which both high and low levels of TSH, FT3, FT4, and conversion ratio values would be associated with incident hypertension. The RRs are presented without adjustment (crude values) and after adjustments for age and sex (Model 1) and multiple variables (Model 2, which included Model 1 plus self-reported race, education level, smoking and alcohol consumption, and physical activity during leisure time). Since BMI may be an important mediator between thyroid function and hypertension, we also analyzed the data comparing groups according to BMI higher or lower than 25 kg/m^2^.

All analyses were performed using the software R, version 3.5.3 (R Core Team, Vienna, Austria). P values < 0.05 were considered statistically significant.

## RESULTS

Table 1 describes the baseline sociodemographic and clinical characteristics of the cohort. The participants were most often women, white, with a high education level (complete college or more), never smokers, who reported current consumption of alcohol. The rate of incident hypertension was 28% (95% CI: 27%-29.3%). When the cohort was stratified based on the development of incident hypertension during follow-up, the participants who developed this complication, compared with those who did not, were more frequently men, black, with a low education level, and with dyslipidemia and diabetes at baseline.

**Table 1:** Sociodemographic and clinical characteristics of the cohort at baseline. The results are shown for the entire cohort and are further stratified based on the development of incident hypertension during an 8.2-year follow-up period

		Incident hypertension		
		All n = 5,915	No n = 4,251	Yes n = 1,664
Age (years), mean (SD)		48.9 (8.18)	48.1 (7.92)	51.1 (8.44)
Women, n (%)		3,304 (55.9%)	2,436 (57.3%)	868 (52.2%)
Race, n (%)				
	White	3,236 (55.2%)	2,389 (56.7%)	847 (51.5%)
	Mixed	1,597 (27.2%)	1,149 (27.2%)	448 (27.2%)
	Black	813 (13.9%)	527 (12.5%)	286 (17.4%)
	Asian	160 (2.73%)	116 (2.75%)	44 (2.67%)
	Indigenous	56 (0.96%)	36 (0.85%)	20 (1.22%)
Education level, n (%)				
	Less than high school	480 (8.11%)	297 (6.99%)	183 (11.0%)
	High school and some college	2,051 (34.7%)	1,431 (33.7%)	620 (37.3%)
	Complete college or more	3,384 (57.2%)	2,523 (59.4%)	861 (51.7%)
Smoking, n (%)				
	Never	3,559 (60.2%)	2,653 (62.4%)	906 (54.4%)
	Past	1,561 (26.4%)	1,052 (24.7%)	509 (30.6%)
	Current	795 (13.4%)	546 (12.8%)	249 (15.0%)
Alcohol consumption, n (%)				
	Never	564 (9.54%)	390 (9.18%)	174 (10.5%)
	Past	1,084 (18.3%)	756 (17.8%)	328 (19.7%)
	Current	4,265 (72.1%)	3,104 (73.0%)	1,161 (69.8%)
Physical activity during leisure time, n (%)			
	Inactive	3,479 (59.7%)	2,428 (58.0%)	1,051 (64.1%)
	Insufficiently active	728 (12.5%)	523 (12.5%)	205 (12.5%)
	Active	1,618 (27.8%)	1,234 (29.5%)	384 (23.4%)
Dyslipidemia, n (%)		3,171 (53.6%)	2,183 (51.4%)	988 (59.4%)
Body mass index (kg/m_2_), mean (SD)		26.0 (4.23)	25.5 (4.05)	27.2 (4.45)
Diabetes, n (%)		546 (9.23%)	303 (7.13%)	243 (14.6%)
TSH (mIU/L), mean (SD)		2.00 (0.82)	2.01 (0.82)	1.96 (0.81)
Free T3 (ng/dL), mean (SD)		0.32 (0.04)	0.32 (0.04)	0.32 (0.04)
Free T4 (ng/dL), mean (SD)		1.19 (0.16)	1.19 (0.15)	1.18 (0.16)
Anti-TPO (IU/mL), mean (SD)		24.3 (59.0)	25.0 (61.8)	22.3 (51.3)
FT3:FT4, mean (SD)		0.27 (0.04)	0.27 (0.04)	0.27 (0.05)
Systolic blood pressure (mmHg), mean (SD)		114 (11.3)	112 (10.6)	121 (10.3)
Diastolic blood pressure (mmHg), mean (SD)		72.6 (8.01)	70.9 (7.53)	76.9 (7.61)

Abbreviations: anti-TPO, thyroid peroxidase antibody; SD, standard deviation.

**Figure 2:**
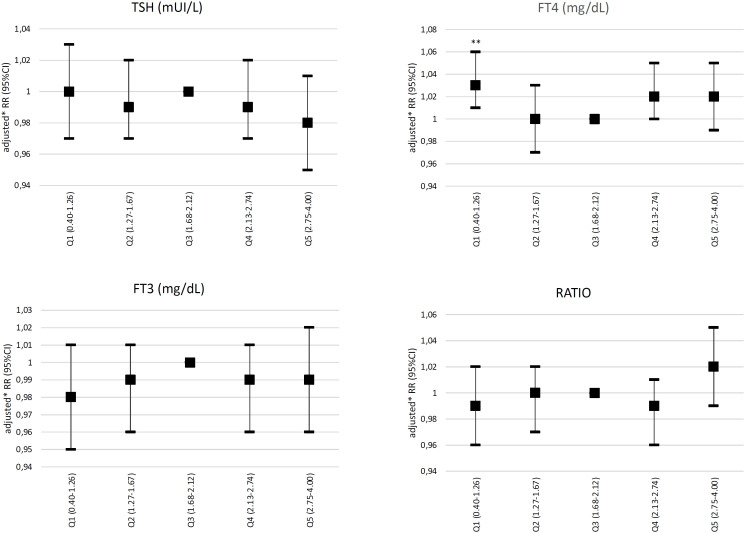
Relative risks of incident hypertension (defined by blood pressure levels ≥ 140/90 mmHg) according to quintiles of TSH, free T4, free T3, and conversion ratio values incidence in euthyroid participants. *Adjustment by age, sex, race, education level, smoking and alcohol consumption, and physical activity. Abbreviations: FT3, free T3; FT4, free T4; Q, quintile(s). **P < 0.05.

**Figure 3:**
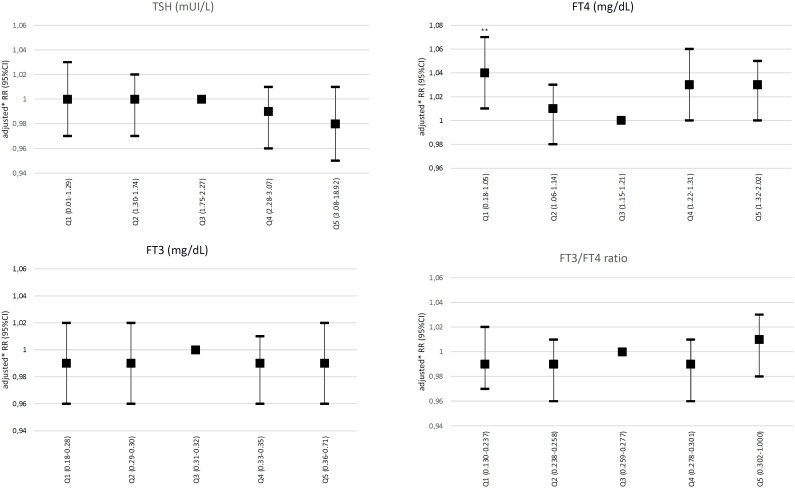
Relative risks of incident hypertension (defined by blood pressure levels ≥ 140/90 mmHg) according to quintiles of TSH, free T4, free T3, and conversion ratio values in combined data of euthyroid participants plus individuals with subclinical thyroid dysfunction. *Adjustment by age, sex, race, education level, smoking and alcohol consumption, and physical activity. Abbreviations: FT3, free T3; FT4, free T4; Q, quintile(s). **P < 0.05.

In the primary analysis incorporating only euthyroid participants, no association was observed between TSH, FT3, or conversion ratio values with incident hypertension (defined by a 140/90 mmHg cutoff value). However, using FT4 levels in the third quintile as a reference, FT4 levels in the first quintile (0.18-1.06 ng/dL) were significantly associated with incident hypertension (adjusted RR: 1.03, 95% CI: 1.01-1.06) (Figure 2). No association between abnormal SBP or DBP values and incident hypertension was observed in the euthyroid subgroup. The association between FT4 levels in the first quintile and incident hypertension was also observed in the secondary analysis incorporating combined data from euthyroid individuals and participants with subclinical thyroid dysfunction (RR: 1.04, 95% CI: 1.01-1.07) (Figure 3). The FT4:FT3 ratio was positively associated with incident hypertension in crude and age- and sex-adjusted models but not in the analysis adjusted for multiple variables.

In the analysis including euthyroid participants and participants with subclinical hypothyroidism, a borderline nonsignificant association was observed between low FT4 levels and hypertension, both when hypertension was defined by SBP levels ≥ 140 mmHg independent of DBP level (RR: 1.02, 95% CI: 1.00-1.04) and by DBP levels ≥ 90 mmHg independent of SBP levels (RR: 1.02, 95% CI: 1.01-1.04).

No associations were observed between TSH, FT4, FT3, or conversion ratio values and incident hypertension when the analysis considered individuals categorized by BMI level (Supplementary Tables 1 and 2).

## DISCUSSION

The main finding of the present study was the mild association between FT4 levels in the first quintile (*i.e.*, in the lowest levels) with incident hypertension, defined as SBP ≥ 140 mmHg and/or DBP ≥ 90 mmHg in euthyroid participants. The association persisted when the analysis included data from euthyroid participants and individuals with subclinical thyroid dysfunction combined. The same analysis also found a borderline association between FT4 levels in the fourth quintile and incident hypertension, resulting in a U-shaped curve, although the same association was not observed in the fifth quintile of FT4 levels. No associations were observed between TSH levels and incident hypertension in the cohort of euthyroid individuals or the combined cohort of euthyroid individuals plus individuals with subclinical thyroid dysfunction.

Analyses looking specifically into alterations in SBP levels independent of DBP levels and vice-versa found that the associations were predominantly observed with SBP levels in euthyroid individuals. However, in the combined analysis incorporating euthyroid participants and individuals with subclinical thyroid dysfunction, the associations were more pronounced with DBP levels. All the associations found in this analysis were mild.

Four prospective studies have evaluated the association of TSH and FT4 levels with incident hypertension, pre-hypertension, and higher SBP and DBP levels in euthyroid individuals. Most of these studies analyzed associations between variations in TSH and FT4 levels and changes in blood pressure levels, while only the study by Abdi and cols. (14) analyzed the association between TSH and FT4 variations with incident pre-hypertension and hypertension. In their study, which included 1,569 participants (women, 55.3%; mean age, about 40 years), the authors found that a 1 ng/dL increase in FT4 levels within the reference range was associated with a 40% increased risk of pre-hypertension but not with increased risk of incident hypertension. These results were observed when the analysis incorporated the entire sample or only men but not when it included only women.

The present study is not the first to find no association between TSH levels and incident hypertension, as the lack of such association has also been reported by Abdi and cols. in a prospective study (14). Park and cols. (13), analyzing a sample of 949 individuals (women, 42.2%; mean age, around 47 years), found that an increase in blood pressure levels from baseline to follow-up was associated with higher TSH levels and that DBP levels were associated with FT4 levels at follow-up. Åsvold and cols. (34) analyzed a sample of 14,353 euthyroid individuals (women, 67.6%; mean age, 51 years) and observed that a 1 mU/L increase in TSH level at baseline was associated with a 0.8 mmHg increase in SBP level and a 0.3 mmHg increase in DBP level over an 11-year follow-up period; these findings were observed only in women and not in men. Jiang and cols. (15) analyzed a sample of 531 individuals (women, 57.6%; mean age, 44.6 years) and observed that a 1 mIU/L increase in TSH levels was associated with about 2 mmHg change in SBP and 1 mmHg change in DBP over a 5-year follow-up period in women but not in men. A recent multicenter study from the Thyroid Studies Collaboration (TSC) has shown a J-shaped association of FT4 levels with cardiovascular disease and mortality; no association between TSH levels and cardiovascular and all-cause mortality was observed, although low TSH levels were found to be associated with these outcomes (35).

Compared with these studies, the present study found an association between low FT4 levels and incident hypertension defined by blood pressure values ≥ 140 and/or ≥ 90 mmHg. The studies by Abdi and cols. (14) and Park and cols. (13) reported an association between increasing FT4 levels and blood pressure. Of note, the present study found that low FT4 levels were associated with incident hypertension, but the study by Abdi and cols. reported that high FT4 levels were associated with pre-hypertension (but not with hypertension). These studies were carried out in middle-income countries and included populations with unique characteristics not analyzed in previous studies. They were both aligned regarding the lack of association between TSH levels and incident hypertension. However, the results of the association between TSH levels and incident hypertension in the present study disagree with the results published by Åsvold and cols. (34) and Jiang and cols. (15), which showed an association between higher TSH levels and higher blood pressure levels.

Two other cohort studies analyzed the association between thyroid function and hypertension in individuals with euthyroidism and subclinical hypothyroidism, one by Ittermann and cols. (17) including 10,048 participants from five studies (women, 45.5-61.9%; age range, 36-74 years) and another by Langén and cols. (16) including 3,453 participants (women, 52.5%; mean age, around 50 years). Unlike the present study, neither found any association between FT4 levels and incident hypertension or increased blood pressure levels. However, their results for TSH levels are similar to those of the present study, *i.e.*, lack of association between TSH levels and incident hypertension.

The present study is the first to analyze the association between the FT3:FT4 ratio and hypertension. The FT3:FT4 ratio indicates the T4 to T3 conversion rate, reflecting the peripheral sensitivity of thyroid hormones. An increased FT3:FT4 ratio may occur due to an adaptation to adverse metabolic conditions with increased type 2 deiodinase (DIO2) activity (36,37) and might be considered an early surrogate marker of TSH and thyroid hormone effects on metabolic parameters (such as obesity and nonalcoholic fatty liver disease) (38) and a predictor of cardiovascular mortality (39). The finding of an increased risk of hypertension with the increasing FT3:FT4 ratio aligns with the association of low FT4 levels with incident hypertension since an increased conversion from FT4 to FT3 decreases the circulating levels of FT4.

Studies analyzing the association between FT3 and hypertension are scarce and have reported conflicting results, either confirming (20) or excluding the association between FT3 levels and high blood pressure (19). The findings of FT3 in our analysis are in accordance with the findings of FT4 levels and FT3:FT4 ratio. Notably, recent evidence has indicated a positive association of FT3 levels with TSH levels and circadian rhythm (37). Also, we could speculate that FT3 may be a more sensitive parameter in conditions requiring adaptation to metabolic adversity, for example, during initial signs of hypertension.

Several reasons could explain the different results between the present study and other similar studies. The ELSA-Brasil multiethnic cohort represents a uniquely diverse sample encompassing individuals of European, African, and native Brazilian descent. This distinctive racial blend is unique and has not been incorporated in prior studies. Also, the outcomes analyzed in each cohort study differed substantially. Two studies analyzed the outcome of the occurrence of incident hypertension (as in the present study) (14,17), while two other studies evaluated the impact of changes in TSH levels on blood pressure levels (15,34). The study by Jiang and cols. analyzed the impact of changes in serum TSH values and variations in blood pressure levels (15). Other possible reasons for the contradicting results include differences in follow-up duration, number of participants in each study, and differences in sex and age distributions. Notably, the results of the present study regarding the association between FT4 level and incident hypertension are aligned with those of a previous analysis of ELSA-Brasil participants investigating the association between TSH, FT4, FT3 levels and the conversion ratio with the incidence of diabetes (34,40). However, an important difference between the two analyses of the ELSA-Brasil study was that BMI was found to have an effect on the association between thyroid hormone levels and the incidence of diabetes but not on the incidence of hypertension.

Our study has some strengths. ELSA-Brasil is a multicentric prospective cohort study conducted in six Brazilian cities. Thyroid function was assessed comprehensively, as it included measurement of TSH, FT4, and FT3 levels and calculation of the conversion ratio. The impact of all the variables on incident hypertension was analyzed in two different ways, one considering a 1-unit increase in TSH, FT3, FT4, and conversion ratio values and the other also considering the levels of hormones stratified by quintiles. The training of the study teams was centralized and conducted under strict quality control supervision (41). The study included a multiethnic cohort from a low-to-middle-income country, and multiethnicity is not well represented in similar articles published on this topic including European (17,34), Asian (15), and Middle Eastern (14) cohorts. The number of women included was slightly greater than that of men, although men were still well represented in the study. The findings had only mild associations, but hypertension is highly prevalent in Brazil and worldwide, so even a modest association may impact health endpoints. In terms of public health, it is critical to raise awareness of the importance of documenting FT4 and TSH levels in patients with cardiovascular risk factors.

The present study also has some limitations. Thyroid hormone and TSH levels were measured only once (at baseline). Since the analysis excluded individuals with overt thyroid diseases, it decreased the likelihood of finding stronger associations. Although blood pressure was measured several times on visit days using standardized protocols, all measurements in the same participant were obtained on a single day. This may represent a limitation in population studies. Finally, since the study found mild associations, the follow-up duration may not have been long enough to show important associations.

In conclusion, low FT4 levels, but not TSH levels, may be a slight risk factor of incident hypertension in euthyroid individuals and persons with subclinical thyroid dysfunction.

## Supplementary Table 1 Association of TSH, free T4, free T3, and conversion ratio values with incident hypertension in euthyroid participants categorized according to body mass index < 25 kg/m^2^ or ≥ 25 kg/m^2^. The variables were analyzed both as continuous values and in quintiles

**Table:**

Incidence of hypertension (≥140/90 mmHg)					
	Range	Events	Crude	Age and Sex	Multivariable**
Body mass index < 25 kg/m^2^					
**TSH**	mIU/L				
Continuous*	-	549 (20.4)	0.98 (0.97-1.00)	0.98 (0.96-0.99)	0.98 (0.97-1.00)
Q1	0.4-1.26	131 (24.3)	1.03 (0.99-1.08)	1.04 (0.99-1.08)	1.03 (0.99-1.07)
Q2	1.27-1.69	110 (20.2)	1.00 (0.96-1.04)	1.00 (0.96-1.04)	1.00 (0.96-1.04)
Q3	1.70-2.12	108 (20.2)	reference	reference	reference
Q4	2.13-2.71	97 (18)	0.98 (0.94-1.02)	0.98 (0.94-1.02)	0.98 (0.94-1.02)
Q5	2.72-4.00	103 (19.3)	0.99 (0.95-1.03)	0.98 (0.95-1.02)	0.99 (0.95-1.03)
**FT4**	ng/dL				
Continuous*	-	549 (20.4)	1.06 (0.97-1.15)	1.05 (0.96-1.14)	1.05 (0.97-1.15)
Q1	0.48-1.08	127 (22.1)	1.03 (0.99-1.07)	1.03 (0.99-1.07)	1.02 (0.98-1.07)
Q2	1.09-1.16	92 (17)	0.99 (0.95-1.03)	0.99 (0.95-1.03)	0.98 (0.95-1.02)
Q3	1.17-1.23	95 (18.4)	reference	reference	reference
Q4	1.24-1.33	122 (22.1)	1.03 (0.99-1.07)	1.03 (0.99-1.07)	1.03 (0.99-1.07)
Q5	1.34-2.02	107 (21.9)	1.03 (0.99-1.07)	1.02 (0.98-1.07)	1.02 (0.98-1.06)
**FT3**	ng/dL				
Continuous*	-	549 (20.4)	1.31 (0.94-1.83)	1.23 (0.87-1.75)	1.18 (0.83-1.68)
Q1	0.18-0.28	120 (19.4)	0.98 (0.94-1.02)	0.99 (0.95-1.03)	0.99 (0.95-1.03)
Q2	0.29-0.30	91 (16.8)	0.96 (0.92-0.99)	0.96 (0.92-1.00)	0.96 (0.92-1.00)
Q3	0.31-0.32	121 (22)	reference	reference	reference
Q4	0.33-0.34	99 (21.5)	1.00 (0.95-1.04)	1.00 (0.96-1.04)	0.99 (0.95-1.04)
Q5	0.35-0.71	113 (22.6)	1.00 (0.96-1.05)	1.01 (0.96-1.05)	1.00 (0.96-1.04)
**FT3:FT4**					
Continuous*	-	549 (20.4)	1.08 (0.76-1.54)	1.03 (0.73-1.46)	0.98 (0.69-1.40)
Q1	0.142-0.233	103 (19.1)	1.00 (0.97-1.04)	1.00 (0.97-1.04)	1.01 (0.97-1.05)
Q2	0.234-0.252	115 (21.7)	1.03 (0.99-1.07)	1.03 (0.99-1.07)	1.02 (0.99-1.07)
Q3	0.253-0.270	100 (18.6)	reference	reference	reference
Q4	0.271-0.292	118 (22.1)	1.03 (0.99-1.07)	1.02 (0.99-1.07)	1.02 (0.98-1.06)
Q5	0.293-0.518	107 (20.2)	1.01 (0.97-1.05)	1.01 (0.97-1.05)	1.01 (0.97-1.05)
**Body mass index ≥ 25 kg/m^2^**					
**TSH**	mIU/L				
Continuous*	-	1115 (34.6)	1.00 (0.98-1.01)	0.99 (0.98-1.01)	1.00 (0.99-1.02)
Q1	0.40-1.26	224 (34.2)	0.98 (0.95-1.02)	0.98 (0.94-1.02)	0.97 (0.93-1.01)
Q2	1.27-1.65	218 (33.8)	0.98 (0.94-1.02)	0.97 (0.94-1.01)	0.98 (0.94-1.02)
Q3	1.66-2.12	236 (36.6)	reference	reference	reference
Q4	2.13-2.76	232 (36.2)	1.00 (0.96-1.04)	0.99 (0.96-1.03)	1.00 (0.96-1.04)
Q5	2.77-4.00	205 (31.9)	0.97 (0.93-1.00)	0.96 (0.92-0.99)	0.97 (0.93-1.00)
**FT4**	ng/dL				
Continuous*	-	1115 (34.6)	0.97 (0.90-1.05)	0.96 (0.89-1.04)	0.98 (0.90-1.06)
Q1	0.18-1.04	235 (34.7)	1.02 (0.98-1.06)	1.01 (0.97-1.05)	1.01 (0.97-1.05)
Q2	1.05-1.13	230 (35.1)	1.02 (0.98-1.06)	1.02 (0.98-1.06)	1.01 (0.97-1.05)
Q3	1.14-1.20	193 (32.7)	reference	reference	reference
Q4	1.21-1.30	234 (35.1)	1.02 (0.98-1.06)	1.01 (0.97-1.05)	1.02 (0.98-1.06)
Q5	1.31-1.93	214 (34.8)	1.02 (0.98-1.06)	1.01 (0.97-1.05)	1.01 (0.97-1.05)
**FT3**	ng/dL				
Continuous*	-	1115 (34.6)	0.87 (0.63-1.20)	0.92 (0.65-1.30)	0.92 (0.65-1.30)
Q1	0.18-0.29	310 (34.8)	0.99 (0.96-1.03)	0.99 (0.96-1.03)	0.99 (0.95-1.02)
Q2	0.30-0.31	243 (35.6)	1.00 (0.96-1.03)	1.00 (0.96-1.04)	1.00 (0.96-1.04)
Q3	0.32-0.33	231 (36.1)	reference	reference	reference
Q4	0.34-0.35	160 (32.1)	0.97 (0.93-1.01)	0.98 (0.94-1.02)	0.98 (0.94-1.02)
Q5	0.36-0.51	163 (32.9)	0.98 (0.94-1.02)	0.98 (0.94-1.03)	0.98 (0.94-1.02)
**FT3:FT4**					
Continuous*	-	1115 (34.6)	1.10 (0.82-1.48)	1.17 (0.87-1.55)	1.10 (0.82-1.46)
Q1	0.130-0.240	236 (36.8)	1.03 (0.99-1.07)	1.01 (0.98-1.05)	1.01 (0.97-1.05)
Q2	0.241-0.262	215 (33.4)	1.00 (0.96-1.04)	0.99 (0.96-1.03)	1.00 (0.96-1.04)
Q3	0.263-0.281	213 (33.3)	reference	reference	reference
Q4	0.282-0.305	204 (31.9)	0.99 (0.95-1.03)	0.99 (0.95-1.03)	0.98 (0.95-1.02)
Q5	0.306-0.299	238 (37.1)	1.03 (0.99-1.07)	1.02 (0.99-1.06)	1.02 (0.98-1.06)

* Continuous variables.

** Adjustment by age, sex, race, education level, smoking and alcohol consumption, and physical activity. Abbreviations: DBP, diastolic blood pressure; FT3:FT4, free T3 to free T4 (conversion) ratio; Q, quintile(s); SBP, systolic blood pressure.

## Supplementary Table 2 Association of TSH, free T4, free T3, and conversion ratio values with incident hypertension in the combined cohort of euthyroid participants and individuals with subclinical thyroid dysfunction categorized according to body mass index < 25 kg/m^2^ or ≥ 25 kg/m^2^. The variables were analyzed both as continuous values and in quintiles

**Table:**

Incident hypertension (≥140/90 mmHg)					
	Range	Events	Crude	Age and Sex	Multivariable**
Body mass index < 25 kg/m^2^					
**TSH**	mIU/L				
Continuous*	-	175 (5.9)	1.00 (0.99-1.00)	0.99 (0.99-1.00)	1.00 (0.99-1.00)
Q1	0.01-1.29	48 (8)	1.02 (1.00-1.05)	1.03 (1.00-1.05)	1.02 (0.99-1.04)
Q2	1.30-1.76	32 (5.5)	1.00 (0.98-1.03)	1.00 (0.98-1.03)	1.00 (0.98-1.02)
Q3	1.77-2.27	32 (5.4)	reference	reference	reference
Q4	2.28-3.06	29 (4.9)	1.00 (0.97-1.02)	0.99 (0.97-1.02)	0.99 (0.97-1.01)
Q5	3.07-14.9	34 (5.8)	1.00 (0.98-1.03)	1.00 (0.97-1.02)	1.00 (0.98-1.03)
**FT4**	ng/dL				
Continuous*	-	175 (5.9)	1.00 (0.9 -1.05)	0.98 (0.93-1.04)	0.98 (0.93-1.04)
Q1	0.49-1.07	38 (6.4)	0.99 (0.97-1.02)	0.99 (0.97-1.02)	0.99 (0.96-1.02)
Q2	1.08-1.15	25 (4.3)	0.97 (0.95-1.00)	0.98 (0.95-1.00)	0.97 (0.95-1.00)
Q3	1.16-1.23	45 (7)	reference	reference	reference
Q4	1.24-1.32	38 (7)	1.00 (0.97-1.03)	1.00 (0.97-1.02)	1.00 (0.97-1.02)
Q5	1.33-2.02	27 (4.7)	0.98 (0.95-1.00)	0.97 (0.95-1.00)	0.97 (0.95-0.99)
**FT3**	ng/dL				
Continuous*	-	175 (5.9)	1.24 (0.98-1.56)	1.14 (0.89-1.45)	1.05 (0.82-1.34)
Q1	0.18-0.28	37 (5.5)	0.99 (0.97-1.02)	1.00 (0.98-1.02)	1.00 (0.97-1.02)
Q2	0.29-0.30	25 (4.2)	0.98 (0.96-1.00)	0.99 (0.96-1.01)	0.99 (0.96-1.01)
Q3	0.31-0.32	37 (6.2)	reference	reference	reference
Q4	0.33-0.34	26 (5.1)	0.99 (0.96-1.01)	0.99 (0.96-1.02)	0.99 (0.96-1.01)
Q5	0.35-0.71	48 (8.7)	1.02 (0.99-1.05)	1.02 (0.99-1.05)	1.01 (0.98-1.04)
**FT3:FT4**					
Continuous*		175 (5.9)	1.24 (0.97-1.59)	1.18 (0.93-1.50)	1.11 (0.87-1.41)
Q1	0.142-0.233	32 (5.5)	1.01 (0.98-1.03)	1.01 (0.98-1.03)	1.01 (0.99-1.03)
Q2	0.234-0.252	34 (5.8)	1.01 (0.99-1.03)	1.01 (0.99-1.04)	1.01 (0.99-1.04)
Q3	0.253-0.271	28 (4.8)	reference	reference	reference
Q4	0.272-0.294	38 (6.5)	1.02 (0.99-1.04)	1.01 (0.99-1.04)	1.01 (0.99-1.04)
Q5	0.295-0.518	41 (7)	1.02 (1.00-1.05)	1.02 (0.99-1.05)	1.01 (0.99-1.04)
**Body mass index ≥ 25 kg/m^2^**					
**TSH**	mIU/L				
Continuous*	-	319 (8.9)	1.00 (0.99-1.01)	1.00 (0.99-1.01)	1.00 (0.99-1.01)
Q1	0.01-1.30	69 (9.6)	1.01 (0.99-1.04)	1.01 (0.99-1.04)	1.01 (0.98-1.03)
Q2	1.31-1.72	74 (10.3)	1.02 (0.99-1.05)	1.02 (0.99-1.05)	1.02 (0.99-1.05)
Q3	1.73-2.27	59 (8.3)	reference	reference	reference
Q4	2.28-3.10	57 (8.1)	1.00 (0.97-1.02)	0.99 (0.97-1.02)	1.00 (0.97-1.02)
Q5	3.11-18.92	60 (8.5)	1.00 (0.98-1.03)	0.99 (0.97-1.02)	1.00 (0.97-1.03)
**FT4**	ng/dL	ng/dL			
Continuous*	-	319 (8.9)	1.01 (0.95-1.07)	0.99 (0.93-1.05)	0.99 (0.93-1.05)
Q1	0.18-1.04	69 (9)	1.02 (0.99-1.05)	1.02 (0.99-1.04)	1.02 (0.99-1.04)
Q2	1.05-1.12	61 (9.3)	1.02 (1.00-1.05)	1.02 (0.99-1.05)	1.03 (1.00-1.06)
Q3	1.13-1.20	51 (6.9)	reference	reference	reference
Q4	1.21-1.30	68 (9.4)	1.02 (1.00-1.05)	1.02 (0.99-1.04)	1.02 (1.00-1.05)
Q5	1.31-1.93	68 (10.4)	1.03 (1.01-1.06)	1.03 (1.00-1.05)	1.03 (1.00-1.05)
**FT3**	ng/dL				
Continuous*	-	319 (8.9)	1.13 (0.91-1.41)	1.06 (0.84-1.35)	1.05 (0.82-1.33)
Q1	0.18-0.29	69 (7.1)	0.98 (0.96-1.01)	0.99 (0.97-1.02)	0.99 (0.97-1.02)
Q2	0.30-0.31	83 (10.9)	1.02 (0.99-1.05)	1.03 (1.00-1.05)	1.02 (1.00-1.05)
Q3	0.32-0.33	63 (8.8)	reference	reference	reference
Q4	0.34-0.35	50 (9.2)	1.00 (0.98-1.03)	1.01 (0.98-1.04)	1.01 (0.98-1.04)
Q5	0.36-0.51	52 (9.4)	1.01 (0.98-1.04)	1.01 (0.98-1.04)	1.00 (0.97-1.03)
**FT3:FT4**					
Continuous*		319 (8.9)	1.10 (0.89-1.35)	1.10 (0.89-1.35)	1.06 (0.86-1.31)
Q1	0.129-0.241	58 (8.2)	1.00 (0.97-1.02)	0.99 (0.97-1.02)	0.99 (0.96-1.02)
Q2	0.242-0.263	74 (10.2)	1.02 (0.99-1.04)	1.01 (0.99-1.04)	1.02 (0.99-1.05)
Q3	0.264-0.282	59 (8.5)	reference	reference	reference
Q4	0.283-0.307	58 (8.2)	1.00 (0.97-1.02)	1.00 (0.97-1.02)	1.00 (0.97-1.02)
Q5	0.308-1.000	68 (9.6)	1.01 (0.98-1.04)	1.00 (0.98-1.03)	1.00 (0.97-1.03)

* Continuous variables.

** Adjustment by age, sex, race, education level, smoking and alcohol consumption, and physical activity. Abbreviations: DBP, diastolic blood pressure; FT3:FT4, free T3 to free T4 (conversion) ratio; Q, quintile(s); SBP, systolic blood pressure.
